# Genotype–phenotype characteristics of Vietnamese patients diagnosed with Charcot–Marie–Tooth disease

**DOI:** 10.1002/brb3.2744

**Published:** 2022-08-08

**Authors:** Trung‐Hieu Nguyen‐Le, Minh Duc Do, Linh Hoang Gia Le, Quynh Nhu Nguyen Nhat, Nghia Trong Tien Hoang, Tuan Van Le, Thao Phuong Mai

**Affiliations:** ^1^ Faculty of Medicine University of Medicine and Pharmacy at Ho Chi Minh City Vietnam; ^2^ Center for Molecular Biomedicine University of Medicine and Pharmacy at Ho Chi Minh City Vietnam; ^3^ 175 Military Hospital Ho Chi Minh City Vietnam

**Keywords:** Charcot–Marie–Tooth disease, genetic mutation, multiplex ligation‐dependent probe amplification, next‐generation sequencing

## Abstract

**Background:**

Charcot–Marie‐Tooth (CMT) disease is one of the most common hereditary neuropathies. Identifying causative mutations in CMT is essential as it provides important information for genetic diagnosis and counseling. However, genetic information of Vietnamese patients diagnosed with CMT is currently not available.

**Methods:**

In this study, we described the clinical profile and determined the mutation spectrum of CMT in a cohort of Vietnamese patients with CMT by using a combination of multiplex ligation‐dependent probe amplification and next‐generation sequencing targeting 11 genes *PMP22, MPZ, EGR2, NEFL, MFN2, GDAP1, GARS, MTMR2, GJB1, RAB7A, LITAF*.

**Results:**

In 31 CMT cases, the mutation detection rate was 42% and the most common genetic aberration was PMP22 duplication. The pedigree analysis showed two de novo mutations c.64C > A (p.P22T) and c.281delG (p.G94Afs*17) in the *NEFL* and *PMP22* genes, respectively.

**Conclusion:**

The results of this study once again emphasize the important role of molecular diagnosis and provide preliminary genetic data on Vietnamese patients with CMT.

## Graphical Abstract

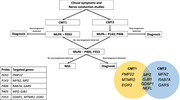

## 1 INTRODUCTION

Charcot–Marie–Tooth (CMT) disease is one of the most common hereditary neuropathies with an estimated prevalence of 1/2500–1/9200 live births (Morena et al., 2019) depending on ethnicity, region, and gender. The typical clinical manifestations of CMT are muscle weakness, atrophy, sensory loss, and reduced musculoskeletal reflex. CMT is traditionally classified into two types, CMT1 (demyelinating) and CMT2 (axonal), based on upper limb motor conduction velocities (MCV) (Pareyson & Marchesi, 2009). The molecular diagnosis of CMT remains challenging because of (i) the diverse phenotypes of a single mutation, (ii) the complex causal genetic spectrum, and (iii) the highly overlapping phenotypes between amyotrophic diseases (Kanwal et al., 2011; Mersiyanova et al., 2000; Pipis et al., 2019; Szigeti & Lupski, 2009). These factors reflect the clinically and genetically heterogeneous nature of CMT. Therefore, molecular diagnosis of CMT is important to patients and their relatives for disease diagnosis and genetic counseling. It is also important to identify the genetic causes of CMT as most novel treatments are gene‐specific. Among the genetic aberrations of CMT, duplication/deletion of PMP22 is the most prevalent, together with pathogenic variants of *MFN2*, *GJB1*, and *MPZ* (Cortese et al., 2020). With advances in DNA sequencing technologies, especially whole‐exome sequencing, the genetic knowledge of CMT is growing, and molecular diagnosis can be made in up to 60% of CMT cases (Bis‐Brewer et al., 2020; Chong et al., 2015; Fridman et al., 2015). However, applying whole‐exome sequencing in clinical practice remains challenging, as this technique is resource‐consuming and coverage is limited. Therefore, customized sequencing panels have been developed for routine genetic tests, and these panels have shown the ability to diagnose up to 30% of CMT cases (Cortese et al., 2020).

With more than 90 million inhabitants, Vietnam is one of the most populated countries in Asia; however, the phenotypic and genetic profiles of CMT in Vietnamese patients have not been investigated in depth. This study aims to examine the clinical characteristics of CMT as well as the diagnostic capability of a genetic analysis combining multiplex ligation‐dependent probe amplification (MLPA) and next‐generation sequencing (NGS) targeting a panel of 11 genes: *PMP22, MPZ, EGR2, NEFL, MFN2, GDAP1, GARS, MTMR2, GJB1, RAB7A*, and *LITAF*.

## 2 MATERIALS AND METHODS

### 2.1 Subjects

The patients were prospectively recruited at the University of Medicine and Pharmacy at Ho Chi Minh City, Vietnam. The CMT diagnosis was made based on clinical signs and symptoms together with a standard electromyography. A total of 31 patients who were diagnosed with CMT agreed to take part in this study, and each patient provided 4 ml of peripheral blood for genetic testing. The clinical profiles of these patients were documented. The protocol for this study was approved by the Ethics Committee of the University of Medicine and Pharmacy at Ho Chi Minh City, Vietnam. Written informed consent was obtained from all patients prior to their enrollment in this study. Once pathogenic mutations were identified in the patients, their first‐degree relatives were also recruited to determine carrier status.

### 2.2 Genetic analysis

#### 2.2.1 Multiplex ligation‐dependent probe amplification

**FIGURE 1:**
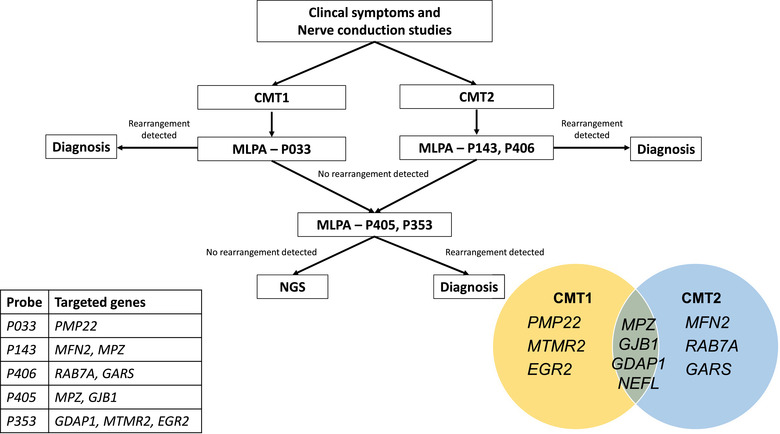
Genetic approach for patients with Charcot–Marie–Tooth (CMT) disease

Genomic DNA was extracted from peripheral leukocytes using Qiagen DNAeasy Blood (Qiagen, Hilden, Germany) according to the manufacturer's protocol. The MLPA reactions were performed using SALSA MLPA Probe‐mix P033(CMT1A), P405(CMTX), P406(CMT2B), P143(CMT2A), and P353(CMT4) (MRC Holland, Amsterdam, the Netherlands). Appropriate probe mixes were chosen based on the guidance for clinical phenotypes; the proposed approach is described in Figure 1. The thermal cycles were carried out according to the manufacturer's instruction. The electropherogram was generated and analyzed using Coffalyser software (MRC Holland).

#### 2.2.2 Targeted NGS

The target NGS test was performed on patients where no genetic rearrangement was detected (Figure 1). The DNA probes for coding regions *PMP22, MPZ, EGR2, NEFL, MFN2, GDAP1, GARS, MTMR2, GJB1, RAB7A*, and *LITAF* were designed and synthesized using xGen Predesigned Gene Capture Pools (Integrated DNA Technologies, IA, United Sates). DNA samples with concentrations equal to or greater than 3.0 ng/μl were fragmented by enzyme to achieve sizes between 100 and 250 bp. NEBNext® Ultra™ II DNA Library Prep Kit for Illumina® (New England Biolabs, MA, United Sates) was used to prepare the sequencing library. The final pooled library was quantified by Qubit 4 Fluorometer (Thermo Fisher Scientific, MA, United States) and subsequently diluted to a final concentration of 2 nM before loading to the sequencing cartridge. The sequencing reactions were performed using a MiniSeq system (Illumina, CA, United States) and a MiniSeq High Output kit (Illumina). The number of samples in each run was calculated to assure 50× or greater coverage for all samples. The online BaseSpace Sequence Hub software developed by Illumina was used to analyze the sequencing results with human genome 19 as the reference. Identified variants were interpreted based on the guidelines of American College of Medical Genetics and Genomics (Richards et al., 2015). The impacts of missense variants were evaluated using Sorting Intolerant From Tolerant (SIFT, http://sift.jcvi.org) and Polymorphism Phenotyping‐2(PolyPhen‐2, http://genetics.bwh.harvard.edu/pph2/) (Adzhubei et al., 2010; Kumar et al., 2009).

#### 2.2.3 Direct sequencing

Direct sequencing was used to confirm the identified pathogenic mutations in the probands and subsequently in first‐degree relatives using appropriately designed primers. The protocol for direct sequencing was described previously (Do et al., 2020, 2021; Kiet et al., 2019).

## 3 RESULTS

### 3.1 Clinical characteristics of patients with CMT

Thirty‐one subjects with CMT disease were recruited for the genetic analysis. The proportion of male and female patients was 1.6:1. Onset was observed in the first two decades of life with motor difficulty (26/31, 83.9%). Most patients exhibited typical clinical phenotypes, including symmetrical distal muscle weakness and atrophy, sensory impairments, decreased or absent deep tendon reflexes, pes cavus and/or joint deformities. Nerve conduction studies helped categorize patients into demyelinating (51.6%), axonal (38.7%), and intermediate CMT (9.7%). The clinical manifestations of patients with CMT are summarized in Table 1.

**TABLE 1:** Clinical and neurophysiological characteristics of patients with Charcot–Marie–Tooth (CMT) disease

	Total (N = 31)	Demyelinated CMT (N = 16)	Axonal CMT (N = 15)
Characteristics and symptoms			
Gender			
Male	19	10	9
Female	12	6	6
Age of onset (years)			
0 to <6	10	4	6
6 to <16	13	6	7
16 to <40	6	6	0
40 to <60	2	0	2
Motor difficulty	25	11	14
Clinical examination			
Reduced distal muscle strength			
Upper limbs	24	12	12
Lower limbs	30	16	14
Distal atrophy			
Upper limbs	18	8	10
Lower limbs	26	14	12
Joint deformity			
Four limbs	9	4	5
Legs	25	9	16
Pes cavus	23	13	10
Sensory impairment			
Upper limbs	19	8	11
Lower limbs	24	12	12
Areflexia			
Upper limbs	25	12	13
Lower limbs	27	13	14
Electromyography examination			
Diagnosis based on nerve conduction velocity			
Demyelinating	16	16	0
Axonal	12	0	12
Intermediate	3	0	3
(Nerve conduction velocity) NCV asymmetrical	12	5	7

*Note*: Axonal CMT included intermediate CMT. Abbreviations: NCV, nerve conduction velocity.

### 3.2 Molecular characteristics of patients with CMT

**FIGURE 2:**
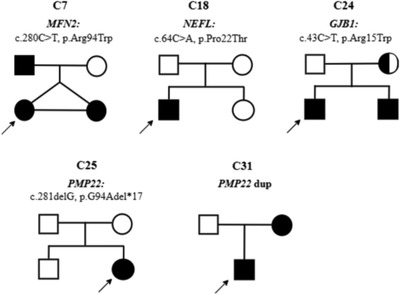
Pedigree analysis of five families with Charcot–Marie–Tooth (CMT) disease harbored confirmed mutations

Genetic testing confirmed the causative gene as CMT1 (56.3%) in nine cases and CMT2 in four cases (26.7%). The molecular detection rate using a combination of MLPA and NGS in this CMT cohort was 41.9% (13 out of 31); in 18 individuals (58.1%), we could not detect any pathogenic alterations within the targeted genes. The most frequent causative gene alteration was *PMP22* (29%), followed by *MFN2* (6.5%), as shown in Table 2. The details of detected point mutations are also described in Table 3. Once mutations were identified in the probands, the corresponding mutations were confirmed in first‐degree relatives by either MLPA or direct sequencing. Five families agreed to take part in pedigree analysis. Two de novo variants, *NEFL* c.64C > A (p.P22T) and *PMP22* c.281delG (p.94Afs*17), were identified in two families. The pedigree analysis is shown in Figure 2. The precise clinical features and electrophysiological data of patients are shown in Table S1.

**TABLE 2:** Distribution of mutations among patients with Charcot–Marie–Tooth (CMT) disease

Genetic aberrations	Clinical phenotype	Percentage of patients (*n* = 31)	Percentage of identified genetic patients (*n* = 13)
PMP22 duplication		25.8 %	61.5 %
	CMT1	8	
	CMT2	0	
	CMT‐I	0	
PMP22 c.281delG (p.G94Afs*17)		3.2 %	7.7 %
	CMT1	0	
	CMT2	1	
	CMT‐I	0	
MFN2 c.280C > T (p.R94W)		6.5 %	15.4%
	CMT1	0	
	CMT2	2	
	CMT‐I	0	
NEFL c.64C > A (p.P22T)		3.2 %	7.7 %
	CMT1	1	
	CMT2	0	
	CMT‐I	0	
GJB1 c.43C > T (p.R15W)		3.2 %	7.7 %
		0	
	CMT1	1	
	CMT2	0	
Unidentifiable	CMT‐I	58.1%	–
	CMT1	7	
	CMT2	8	
	CMT‐I	3	

**TABLE 3:** Characteristics of detected point mutations

Gene	dbSNP	Mutation		SIFT score	Polyphen‐2 score	ExAc frequency (Asian)	ACMG classification	Phenotype
		cDNA	Protein					
*PMP22*	rs80338763	c.281delG	(p.G94Afs*17)	n/a	n/a	n/a	Pathogenic	CMT1E
*MFN2*	rs119103263	c.280C > T	(p.R94W)	0.00	1.000	0	Pathogenic	CMT2A
				Affect protein function	Probably damaging			
*NEFL*	rs28928910	c.64C > A	(p.P22T)	0.04	0.951	0	Pathogenic	CMT1F
				Affect protein function	Possibly damaging			
*GJB1*	rs116840815	c.43C > T	(p.R15W)	0.00	1.000	0	Pathogenic	CMT1X
				Affect protein function	Probably damaging			

Abbreviations: ACMG, American College of Medical Genetics and Genomics; Polyphen‐2, polymorphism phenotyping‐2; SIFT, sorting intolerant from tolerant.

#### 3.2.1 PMP22 duplication

Eight unrelated neuropathy patients had heterozygous *PMP22* duplication. Age at the onset of the disease ranged from 6 up to 40 years old. Clinical phenotypes of these patients were diverse, with frequent symptoms including mild or moderate paresis and atrophy of upper and/or lower limbs, joint deformity, and reduced or absent tendon reflex. Interestingly, there was significantly worse distal limb atrophy in the early‐onset group compared with the late‐onset group. Electrophysiology registered severe demyelinating neuropathy with slow motor nerve conduction and diminished sensory response in all patients. A family history of motor deficits was noted in five subjects (C1, C2, C3, C29, and C31). The pedigree and genotype of one family with PMP22 duplication were confirmed (Figure 2).

#### 3.2.2 PMP22 c.281delG (p.G94Afs*17) variant

An 11‐year‐old girl (C25) suffered from symmetric mild weakness and distal atrophy in her legs, loss of all sensations, and pes cavus. These symptoms slowly progressed and worsened, started from age 2. There was no evidence of hearing impairment or CNS deficit. No response could be detected on investigated nerves. From a combination of clinical and electromyographic analysis, axonal CMT was clinically proposed. The proband's parents exhibited no symptom of neuropathological disorder. A single‐nucleotide heterozygous deletion, c.281delG (p.94Afs*17), was found in the proband.

#### 3.2.3 NEFL c.64 C > A (p.P22T) variant

The proband (C18) was a 24‐year‐old man who presented with a juvenile‐onset motor and sensory neuropathy. At the time of examination, C18 showed symmetrical distal atrophy and loss of all sensations and tendon reflexes, as well as exhibiting pes cavus and joint deformities in the upper and lower limbs. Electrophysiological diagnostics confirmed demyelinating CMT1 affecting motor conduction in the upper limbs, while no response was recorded in lower limb motor conduction or sensory velocities.

#### 3.2.4 MFN2 c.280C > T (p.R94W) variant

Two unrelated patients (C7, C19) harbored *MFN2* mutation p.R94W and shared similarities in early‐age onset, mild reduced muscle strength, and areflexia. NCS suggested axonal neuropathy.

Patient C7 and her twin sister presented identical atrophy of distal legs, pes cavus, and foot deformity, while sensation was intact. Electrophysiological findings on the upper limbs recorded low compound muscle action potential (CMAP) and normal distal motor latency (DML) and MCV; no responses could be detected in sensory and lower limbs motor conduction. DNA testing revealed a heterozygosity of *MFN2* c.280C > T in the proband, her sibling, and father.

Patient C19 suffered atrophy of the lower limbs and foot deformity. Sensory impairment was not identical in the upper and lower extremities: asymmetrical loss of pain as well as vibration were evident in the lower limbs, while the upper limbs remain intact. Patterns of electromyography exhibited no response for motor and sensory conduction in the lower limbs, except for low CMAP on the bilateral posterior tibial nerves. Sensorimotor conduction of the upper limbs remained in the normal range.

#### 3.2.5 GJB1 c.43C > T (p.R15W) variant

A 39‐year‐old male patient (C24) initially exhibited symptoms as an adolescent. At the age of 37, moderate symmetrical atrophy was evident in the upper and lower limbs, along with pes cavus, joint deformities, reduced sensations in the upper and lower extremities, and areflexia. CMAP and motor nerve conduction of the bilateral ulnar nerves were moderately reduced, while motor and sensory conduction of other nerves were preserved. The patient's mother and younger brother expressed a milder form of CMT. We identified the hemizygosity of c.43C > T nucleotide substitution in *GJB1* in the proband, his mother and his sibling, while the father was asymptomatic. A confirmed diagnosis of CMT1X is established in this family.

#### 3.2.6 Unidentified molecular testing

Genetic cause was not confirmed in more than half of patients with obvious family history and clinical criteria of sensorimotor neuropathies. Interestingly, diverse disease severity and asymmetrical conduction features were observed in most of genetically unidentifiable patients.

## 4 DISCUSSION

Nerve conduction studies have been crucial in the classification of forms of CMT since the first electrophysiological diagnostic schemes were proposed in 1968 by Dyck and Lambert (Dyck et al., 1989). The prevalence of CMT is estimated at between 1/2500 and 9.7/100000 (Barreto et al., 2016; Skre, 1974), varying between races. Molecular testing rates range from 25% to 60% (Chong et al., 2015; Fridman et al., 2015), with a higher percentage of CMT1 patients receiving a genetic diagnosis (37.7%–91.2%) than CMT2 and intermediate CMT cases (17%–43.2%) (Bis‐Brewer et al., 2020). Our data show an overall diagnosis rate of 41.9% (13/31 patients), with 56.3% of CMT1 cases and 26.7% of CMT2 cases being solved.

*PMP22* mutants, especially heterozygous duplication of *PMP22*, are strongly related to autosomal dominant CMT1A (Lupski, 1992; Timmerman et al., 1990). PMP22 has an important function in compromising the myelin structure. Our study found *PMP22* variants, including duplication and point mutation, accounting for 69.2% of the identified genetic patients and 29% with sensorimotor neuropathy, comparable to previous studies (Bis‐Brewer et al., 2020). The phenotype in patients with *PMP22* duplication manifests classic motor and sensory deficit symptoms. No relationship could be established when analyzing the age of onset, sex, disease progression, clinical manifestations, electrophysiological characteristics, or the existence of variable lengths of DNA within chromosome 17p11.2 containing the *PMP22* gene. Additionally, we described a patient (C25) manifesting the nerve conduction pattern of axonal CMT with a point mutation in *PMP22* gene. Molecular testing found a de novo alteration located in the last exon of *PMP22* (exon 5), c.281delG (G94Afs). Exon 5 encodes the remaining half of the third transmembrane domain, the second extracellular domain, the fourth transmembrane domain, and the 3′ untranslated region (Li et al., 2013). It was supposed that the deletion of nucleotide G at position 281 led to a premature termination codon, switching categorization to CMT1E—a very rare subtype of CMT1 (Bird et al., 1993). Patients with R159C mutation in *PMP22* have also been reported as presenting mild to severe axonal CMT1E (Gess et al., 2011). Thus, *PMP22* should be investigated cautiously in connection with axonal as well as demyelinating CMT.

To date, point mutations on *PMP22* related to CMT1E have been documented including, 44 missense mutations, 14 deletions, two insertions, one reciprocal translocation, and several splice‐site and single base substitutions in noncoding region 3′ UTR (Li et al., 2013). Three cases with the same 1‐bp deletion in codon 94 of *PMP22* have been published (Boerkoel et al., 2002; Ionasescu et al., 1997; Niedrist et al., 2009). Our proband diagnosed with a hereditary motor and sensory neuropathy at an early age shared comparable severe clinical phenotypes with the reported cases, although spine deformity and deafness were not detected (Table 4). It is obvious that the CMT1E phenotype resulted from the deletion c.281delG (G94Afs) is more severe than the classical CMT1A phenotype caused by the duplication in PMP22. Although G94Afs has been determined to be pathogenic, it is not clear how the mutant leads to variable phenotypes, or aberrant structural and functional truncated proteins.

**TABLE 4:** Comparison of patients with PMP22 (c.281delG, p.G94Afs*17) mutation

	Our patient (C25)	Niedrist et al. (2009)	Boerkoel et al. (2002)	Ionasescu et al. (1997)
Clinical diagnosis	CMT2*	CMT1	Dejerine–Sottas syndrome	CMT1
Inheritance pattern	Sporadic	Sporadic	Sporadic	N/A
Sex	Female	Male	Female	Male
Current age (years)	11	20	27	27
Age at onset (years)	2	1	4	1
Motor impairment	Moderate, distal	Severe, distal	Severe, distal	Severe, distal
Joint deformity	Yes	Yes, pes cavus	Yes, pes cavus	N/A
Deep tendon reflex	Absent	Absent	Absent	Absent
Sensory disturbance	Reduced	Absent	Absent	Absent
Cranial nerve involvement	No	No	Yes	Yes
Gait disturbance	No	Yes	Yes	Yes
Spine	Normal	Progressive scoliosis	Thoracic scoliosis	N/A
Motor nerve conduction ^(median or ulnar)^	Not measurable	Not measurable	4.4 m/s	15–25 m/s, respectively

* Clinical diagnosis was based on the nerve conduction testing at the time that patient was recruited in the study.

Neurofilament light chain polypeptide (NEFL) is abundant in the cytoskeletal structure of neurons (Brownlees et al., 2002). Codon 22 is highly conserved in the head domain among species and seems to be one of the mutational hot spots of *NEFL* (Shin et al., 2008). Three types of nucleotide change in position 64 in exon 1 (64C > T, 64C > A, 64C > G) leading to amino acid substitutions (Pro22Ser, Pro22Thr, Pro22Arg) and have been reported in patients with CMT. These changes may affect NEFL phosphorylation and result in different clinical presentations of demyelinating, axonal, or intermediate CMT (De Jonghe et al., 2001; Georgiou et al., 2002; Jordanova et al., 2003; Shin et al., 2008; Yoshihara et al., 2002). Pro22Thr was first described in a Japanese family with three affected generations (Yoshihara et al., 2002). We compared phenotypes of our patient manifesting Pro22Thr mutation with those of previously reported mutations in Pro22 patients (Table 5). Genotype–phenotype correlations have not yet been established. Our report emphasizes the marked variable phenotypes among patients with CMT comprising mutations at the same codon as NEFL mutations.

**TABLE 5:** Characteristics of patients with mutations on the Pro22 in *NEFL* gene

	Pro22Thr	Pro22Ser	Pro22Arg	
	Our patient (C18)	Yoshihara et al. (2002)	Georgiou et al. (2002) Fabrizi et al. (2004)	Shin et al. (2008)
Clinical diagnosis	CMT1	CMT1	CMT2	CMT1
Inheritance pattern	Sporadic	AD	AD	AD
Age at onset (years)	6 to <16	18–24	<10	3–13
Muscle weakness	Mild/moderate	Moderate	Mild/moderate	Moderate/Severe
Muscle atrophy	UL = LL	UL < LL	UL < LL	UL < LL
Sensory loss	Yes	Yes	Yes	Yes
Pes cavus	Yes	N/A	Yes	Yes
Tendon reflex	Absent	N/A	Reduced/Absent	Absent
Median MCV (m/s)	24–29.7	29–36	21–54	22–29
Median CMAP (mV)	1.6–3.8	0.01–0.74	0.8–4.6	0.1–3.7
Median SCV (m/s)	Not measurable	24	Not measurable	21–27
Median SNAP (μV)	Not measurable	0.7	Not measurable	4.2–5.3

Abbreviation: MCV, motor conduction velocities; CMAP, compound muscle action potential; SCV, sensory conduction velocities; SNAP, sensory nerve action potential.

The Mitofusin 2 (*MFN2*) gene encodes an outer mitochondrial membrane protein that is fundamental to mitochondrial fusion (Dorn, 2020). Mutations in *MFN2* relate to the genetic basis of the CMT2A phenotype and account for 14%–29% of neuropathy patients depending on ethnicity (Bergamin et al., 2014; Calvo et al., 2009; Casasnovas et al., 2010; Kijima et al., 2005; Lin et al., 2011; Szigeti et al., 2006; Züchner et al., 2004). Our study showed that the frequency of the *MFN2* mutation is approximately 6.5% of the patients with CMT and 13.3% of axonal patients with CMT, similar to other studies (Braathen et al., 2011; Verhoeven et al., 2006). Substitution of nucleotide C at position 280 at exon 4 could be either c.280C > T or c.280C > A, leading to missense mutations at codon 94 (R94W and R94Q, respectively). These mutations have been detected in several populations of CMT2 families (Chung et al., 2006; Züchner et al., 2006, 2004). This position in the GTPase domain is highly conserved and necessary for mitochondrial membrane fusion, tethering to the endoplasmic reticulum (Choi et al., 2015; Dorn, 2020). Codon 94 is also among high frequency mutational spots of *MFN2*, in addition to codon 105, 280, and 364. Two unrelated mutational R94W axonal patients with CMT (C7, C25) demonstrated early‐age onset, moderate motor impairment characterized by distal muscle weakness predominating in the lower limbs, decreased deep tendon reflexes, foot deformities, and severe sensory deficits in distal limbs; no visual dysfunction was found. Autosomal dominant inheritance was unequivocally observed in one patient (C7) as previously reported (Kijima et al., 2005; Züchner et al., 2004). While homozygous Mfn2^R94W^ in mice is lethal at birth, heterozygous Mfn2^R94W^ has been shown to lead to the development of mild axonal peripheral neuropathy, which can mimic the model for CMT2A patients (Strickland et al., 2014).

Gap junction protein beta‐1 connexin 32(GJB1, Cx32) participates in the formation of intercellular gaps distributed in Schwann cells, oligodendrocytes and even non‐neural tissue (Dermietzel et al., 1990). *GJB1* mutations cause the second most common type of CMT, the X‐linked CMT disease (CMT1X). More than 450 different alterations in *GJB1* including missense, frameshift, deletion, nonsense mutations, and mutations in noncoding regions, have been published. Mutated p.R15W is located on the N‐terminal region of the molecule involved in the response to trans‐junctional voltage (Abrams et al., 2002; Verselis et al., 1994), causing loss of functional gap junction channels (Abrams et al., 2002). The clinical phenotype of R15W patients in our study exhibited hereditary motor and sensory neuropathy; it was not possible to use an electrophysiological study to differentiate primarily axonal or demyelination damage. The frequency of *GJB1* mutations in this study is lower than previous studies in Asian as well as American and European countries (Liu et al., 2020).

This study demonstrated the genotypic and phenotypic profiles of 31 unrelated patients with CMT. Previously, molecular diagnosis in Vietnam of CMT was performed mostly using consecutive direct sequencing and MLPA. This approach is time‐consuming and cannot be used for large genes. Advances in NGS have provided a platform to overcome these barriers, and NGS has been widely applied in various fields (Do et al., 2020, 2022; Nguyen et al., 2021, 2020). Using targeted panel sequencing of the most frequently reported genes in CMT (*PMP22, MPZ, EGR2, NEFL, MFN2, GDAP1, GARS, MTMR2, GJB1, RAB7A, LITAF*) enabled us to identify approximately 48% of the clinically diagnosed patients with CMT. In most of the tested genes, causal mutations were found in only a single patient or family. From our first report on a novel *GDAP1* mutation (Mai et al., 2019), these results provide additional data to enrich genotype–phenotype correlation in Vietnamese patients with CMT, compared to other ethnicities. Patients with identical mutations could either share common clinical characteristics or exhibit diverse manifestations. Molecular genetic diagnosis plays an important role in diagnosing hereditary motor and/or sensory neuropathies, including CMT disease, in combination with clinical examination. This combination creates a rich scenario for the treatment of these diseases and emphasizes the need to identify the cause using current knowledge and develop methodologies for understanding potential therapeutics for CMT.

## CONFLICT OF INTEREST

The authors declare no conflict of interest.

## AUTHOR CONTRIBUTIONS

Thao Phuong Mai, Minh Duc Do, and Trung‐Hieu Nguyen‐Le designed the study. Trung‐Hieu Nguyen‐Le, Nghia Trong Tien Hoang, Tuan Van Le, and Thao Phuong Mai recruited the patients and analyzed clinical data. Minh Duc Do, Quynh Nhu Nguyen Nhat, and Linh Hoang Gia Le performed genetic testing. Thao Phuong Mai and Minh Duc Do wrote the manuscript. The manuscript was critically revised and approved by all the authors.

### PEER REVIEW

The peer review history for this article is available at: https://publons.com/publon/10.1002/brb3.2744.

## Supporting information

Supplementary Table 1. Clinical features and neurophysiologic data of CMT patients.

## Data Availability

The datasets used and/or analyzed during the current study are available from the corresponding author on reasonable request.
