# Supplementary material for: Genotype–phenotype characteristics of Vietnamese patients diagnosed with Charcot–Marie–Tooth disease
Source: Brain Behav. 2022 Aug 8;12(9):e2744. doi: 10.1002/brb3.2744 (PMC9480926; doi:10.1002/brb3.2744)
Supplement: Supplementary file 1 — Supplementary Table 1. Clinical features and neurophysiologic data of CMT patients. [file BRB3-12-e2744-s001.docx]

**Supplementary Table 1. Clinical features and neurophysiologic data of CMT patients.**

| **Genetic diagnosis** | **Patient ID** | **CMT type** | **Gender** | **Age at examination (yrs)** | **Age of onset**  **(yrs)** | **Muscle strength** | | **Muscle atrophy** | | **Joint defor-mity / Pes cavus** | **Sensory** | **Tendon reflex** | **Nerve conduction studies** | | | | | |
| --- | --- | --- | --- | --- | --- | --- | --- | --- | --- | --- | --- | --- | --- | --- | --- | --- | --- | --- |
|  |  |  |  |  |  |  |  |  |  |  |  |  | **DML** | **MCV** | **CMAP** | **DSL** | **SCV** | **SNAP** |
|  |  |  |  |  |  | **UL**  **D/P** | **LL**  **D/P** | **UL**  **D/P** | **LL**  **D/P** |  | **UL/LL** | **UL/LL** | **UL/LL** | **UL/LL** | **UL/LL** | **UL/LL** | **UL/LL** | **UL/LL** |
| **PMP22 duplication** | **C1** | **CMT 1** | M | 57 | 16-<40 | 3/5 | 1/5 | -/- | +/+ | +/+ | ++/- | -/- | +++/- | +++/- | ++/- | -/- | -/- | -/- |
|  | **C2** | **CMT 1** | F | 37 | 16-<40 | 5/5 | 3/5 | -/- | -/+ | -/- | ++/++ | +/- | ++/- | ++/- | ++/- | -/- | -/- | -/- |
|  | **C3** | **CMT 1** | F | 35 | 16-<40 | 5/5 | 4/5 | -/- | -/- | -/- | +/+ | +/++ | ++/- | ++/- | ++/- | -/- | -/- | -/- |
|  | **C4** | **CMT 1** | M | 39 | 16-<40 | 3/5 | 1/5 | -/- | +/+ | +/+ | ++/- | -/- | ++/- | ++/++ | ++/++ | -/- | -/- | -/- |
|  | **C6** | **CMT 1** | F | 57 | 16-<40 | 4/4 | 3/4 | -/- | +/- | -/- | +/++ | +/++ | ++/- | ++/- | ++/++ | -/- | -/- | -/- |
|  | **C21** | **CMT 1** | M | 22 | 6-<16 | 4/5 | 3/4 | +/- | +/+ | +/+ | ++/++ | -/- | ++/++ | +++/  +++ | ++/++ | -/- | -/- | -/- |
|  | **C29** | **CMT 1** | F | 32 | 6-<16 | 4/4 | 4/4 | +/- | +/- | +/+ | +/+ | -/+ | +++/- | ++/- | ++/- | -/- | -/- | -/- |
|  | **C31** | **CMT 1** | M | 13 | 6-<16 | 4/5 | 4/4 | +/- | +/- | +/+ | +/++ | -/- | ++/++ | +++/  +++ | +/++ | -/- | -/- | -/- |
| **NEFL c.64C>A (p.P22T)** | **C18** | **CMT 1** | M | 24 | 6-<16 | 4/4 | 3/4 | +/- | +/- | ++/+ | -/- | -/- | ++/- | ++/- | ++/- | -/- | -/- | -/- |
| **PMP22 c.281delG (p.G94Afs*17)** | **C25** | **CMT 2** | F | 11s | 0-<6 | 4/4 | 3/3 | +/- | +/- | ++/+ | ++/++ | -/- | -/- | -/- | -/- | -/- | -/- | -/- |
| **MFN2 c.280C>T (p.R94W)** | **C7** | **CMT 2** | F | 8 | 0-<6 | 4/4 | 3/4 | -/- | +/- | +/+ | +/+ | -/- | +/- | ++/- | ++/- | -/- | -/- | -/- |
|  | **C19** | **CMT 2** | F | 8 | 0-<6 | 4/5 | 3/4 | -/- | +/+ | +/- | +/++ | -/- | +/- | +/- | +/++ | +/- | +/- | +/- |
| **GJB1 c.43C>T (p.R15W)** | **C24** | **CMT 2** | M | 39 | 6-<16 | 4/5 | 4/5 | +/- | +/- | ++/+ | ++/++ | -/- | +/- | ++/- | ++/- | -/- | -/- | -/- |

**UL**: upper limb; **LL**: lower limb; **D/P**: distal/proximal; **Muscle strength** was evaluated on scale of 5 degree; **Muscle atrophy, Pes cavus**: + Yes, - No; **Joint deformity**: + foot only, ++ hands and legs, - No; Sensory: + normal, ++ reduced, - loss; **Tendon reflex**: + normal, ++ reduced, - absent; **DML/ DSL**: + normal range, ++ prolonged, - diminished; **MCV/SCV**: + normal, ++ moderate reduced, +++ severe reduced , - absent; **CMAP/SNAP**: + normal, ++ moderate reduced, +++ severe reduced, - absent; Asym.: asymmetrical

| **Genetic diagnosis** | **Patient ID** | **CMT type** | **Gender** | **Age at examination (yrs)** | **Age of onset**  **(yrs)** | **Muscle strength** | | **Muscle atrophy** | | **Joint defor-mity / Pes cavus** | **Sensory** | **Tendon reflex** | **Nerve conduction studies** | | | | | | | |
| --- | --- | --- | --- | --- | --- | --- | --- | --- | --- | --- | --- | --- | --- | --- | --- | --- | --- | --- | --- | --- |
|  |  |  |  |  |  |  |  |  |  |  |  |  | **DML** | **MCV** | **CMAP** | **DSL** | **SCV** | | **SNAP** | |
|  |  |  |  |  |  | **UL**  **D/P** | **LL**  **D/P** | **UL**  **D/P** | **LL**  **D/P** |  | **UL/LL** | **UL/LL** | **UL/LL** | **UL/LL** | **UL/LL** | **UL/LL** | **UL/LL** | | **UL/LL** | |
| **Unconfirmed** | **C5** | **CMT INT.** | M | 7 | 6-<16 | 5/5 | 4/5 | -/- | -/- | -/- | -/- | +/+ | +/+ | +/+ | -/+ | +/+ | | +/+ | | +/+ |
|  | **C8** | **CMT 1** | M | 53 | 16-<40 | 4/4 | 3/4 | +/- | +/- | ++/+ | ++/++ | -/- | +/- | +++/- | ++/-  Asym. | +++/- | | ++/- | | ++/- |
|  | **C9** | **CMT 1** | M | 7 | 0-<6 | 5/5 | 4/5 | -/- | +/- | +/+ | +/+ | -/- | ++/++ | +++/  +++ | ++/++  Asym. | +++/- | | +++/- | | ++/- |
|  | **C10** | **CMT 1** | M | 10 | 6-<16 | 5/5 | 4/5 | -/- | +/- | +/+ | +/+ | +/- | +/- | +/- | ++/-  Asym.. | +/- | | +/- | | +/- |
|  | **C11** | **CMT 2** | M | 17 | 6-<16 | 4/5 | 4/4 | +/- | +/- | +/+ | ++/++ | -/- | ++/- | ++/- | +++/- | -/- | | -/- | | -/- |
|  | **C12** | **CMT 1** | F | 1 | 0-<6 | 2/2 | 1/1 | +/- | +/- | ++/+ | N/A | -/- | ++/-  (++ R.  median n.) | +++/-  (+++R.  median n.) | ++/-  (++R.  median n.) | -/- | | -/- | | -/- |
|  | **C13** | **CMT 2** | M | 13 | 6-<16 | 4/4 | 4/4 | +/+ | +/+ | ++/+ | -/++ | -/- | +/- | +/- | ++/- | +/- | | +/- | | ++/- |
|  | **C14** | **CMT 1** | M | 12 | 6-<16 | 4/4 | 4/4 | +/- | +/+ | +/+ | -/++ | -/- | ++/++ | +++/  +++ | ++/++ | -/- | | -/- | | -/- |
|  | **C15** | **CMT 1** | F | 13 | 0-<6 | 4/4 | 3/4 | -/- | +/+ | +/+ | +/++ | -/- | ++/- | +++/- | ++/-  Asym.. | -/- | | -/- | | -/- |
|  | **C16** | **CMT 2** | M | 13 | 0-<6 | 4/4 | 4/4 | +/+ | +/+ | +/+ | -/++ | -/- | +/+ | ++/++ | +/+ | -/- | | -/- | | -/- |
|  | **C17** | **CMT 2** | M | 14 | 6-<16 | 4/5 | 4/5 | +/- | +/- | +/+ | ++/++ | -/- | +/-  (- Peroneal n.) | ++/-  (- Peroneal n.) | ++/-  (- Peroneal n.) | -/- | | -/- | | -/- |
|  | **C20** | **CMT 2** | M | 36 | 6-<16 | 4/4 | 3/4 | +/+ | +/+ | ++/+ | ++/++ | -/- | ++/-  Asym. | ++/-  Asym. | ++/-  Asym.. | -/- | | -/- | | -/- |
|  | **C22** | **CMT INT.** | F | 25 | 6-<16 | 5/5 | 4/5 | +/- | +/- | +/+ | +/++ | -/- | ++/-  (- Peroneal n.) | ++/-  Asym. | ++/-  Asym. | ++/-  Asym. | | ++/-  (++ R.  median n.) | | ++/-  (++ R.  median n.) |
|  | **C23** | **CMT 2** | F | 1 | 0-<6 | 3/4 | 3/4 | -/- | +/- | +/- | N/A | -/- | +/-  (-L.tibial n.) | -/- | ++/-  (-L.tibial n.) | -/- | | -/- | | -/- |
|  | **C26** | **CMT 2** | M | 61 | 40-<60 | 4/4 | 5/4 | +/- | -/- | -/- | -/++ | -/- | +/+ | ++/+  Asym. | ++/-  Asym. | +/++  Asym. | | +/+++  Asym. | | +/++  Asym. |
|  | **C27** | **CMT 2** | M | 7 | 0-<6 | 5/5 | 4/4 | -/- | -/- | -/- | ++/++ | +/- | ++/+  (++ R.  median n.) | ++/+  Asym. | +/++ | +/++  Asym. | | ++/-  Asym. | | +/-  Asym. |
|  | **C28** | **CMT INT.** | F | 61 | 40-<60 | 2/4 | 3/4 | +/- | +/- | ++/+ | ++/++ | -/- | ++/-  Asym. | ++/-  Asym. | +/++  Asym. | +/++  Asym. | | +/+++  Asym. | | ++/-  Asym. |
|  | **C30** | **CMT 1** | M | 23 | 0-<6 | 3/4 | 3/4 | +/+ | +/+ | ++/+ | ++/++ | -/- | ++/- | +++/- | ++/- | -/- | | -/- | | -/- |

**UL**: upper limb; **LL**: lower limb; **D/P**: distal/proximal; **Muscle strength** was evaluated on scale of 5 degree; **Muscle atrophy, Pes cavus**: + Yes, - No; **Joint deformity**: + foot only, ++ hands and legs, - No; Sensory: + normal, ++ reduced, - loss; **Tendon reflex**: + normal, ++ reduced, - absent; **DML/ DSL**: + normal range, ++ prolonged, - diminished; **MCV/SCV**: + normal, ++ moderate reduced, +++ severe reduced , - absent; **CMAP/SNAP**: + normal, ++ moderate reduced, +++ severe reduced, - absent; Asym.: asymmetrical
